# High-resolution analysis of aberrant regions in autosomal chromosomes in human leukemia THP-1 cell line

**DOI:** 10.1186/1756-0500-2-153

**Published:** 2009-07-27

**Authors:** Naoki Adati, Ming-Chih Huang, Takahiro Suzuki, Harukazu Suzuki, Toshio Kojima

**Affiliations:** 1 Computational Systems Biology Research Group, RIKEN Advanced Science Institute, 1-7-22 Suehiro-cho, Tsurumi-ku, Yokohama, Kanagawa 230-0045, Japan; 2 RIKEN Omics Science Center, 1-7-22 Suehiro-cho, Tsurumi-ku, Yokohama, Kanagawa 230-0045, Japan; 3 Current address : Department of Biological Sciences and Technology, National University of Tainan, 33, Sec. 2, Shu-Lin St., Tainan 700-05, Taiwan, Republic of China

## Abstract

**Background:**

THP-1 is a human monocytic leukemia cell line derived from a patient with acute monocytic leukemia. The cell line differentiates into macrophage-like cells by stimulation with phorbol myristate acetate (PMA). Although it has been used frequently as a model for macrophage differentiation in research including the FANTOM4/Genome Network Project, there are few reports on its genomic constitution. Therefore, we attempted to reveal the genomic aberrations in these cells with the microarray-based comparative genomic hybridization (aCGH) technique.

**Findings:**

We report large aberrations, including deletions 6p, 12p, 17p, and trisomy 8, and revealed breakpoints in the *MLL *and *MLLT3 *genes. Moreover, we found novel genomic aberrations such as a hemizygous narrow deletion partially containing the *TP73 *gene and homozygous deletions, including the *CDKN2A*, *CDKN2B *and *PTEN *genes.

**Conclusion:**

In this study, we identified 119 aberrant regions in autosomal chromosomes, and at least 16 of these aberrations were less than 100 kb, most of which were undetectable in the previous works. We also revealed a total of 4.6 Mb of homozygous deleted regions. Our results will provide a base to precisely understand studies involving the THP-1 cell line, especially the huge amount of data generated from the FANTOM4/Genome Network Project.

## Introduction

As models for the study of myeloid differentiation and hematopoietic cell differentiation, several human leukemia cell lines are available [1]. Although these myeloid leukemia cell lines are blocked at certain steps in the maturation and differentiation process, they can be induced to differentiate into macrophage-like cells by several stimuli [1,2].

THP-1 is a human monocytic leukemia cell line that was cultured from the blood of a 1-year-old male with acute monocytic leukemia [3]. On stimulation with phorbol 12-myristate 13-acetate (PMA), THP-1 cells cease proliferation, become adherent, and differentiate into macrophage-like cells. They resemble native monocyte-derived macrophages with respect to numerous criteria [4,5]. In comparison with other human myeloid cell lines such as HL-60, U937, KG-1 or HEL cells, differentiated THP-1 cells behave more like native monocyte-derived macrophages [5]. Because of these characteristics, the THP-1 cell line is a valuable model for studying the mechanisms involved in macrophage differentiation. Therefore, THP-1 has been used not only as a clinical model of a leukemic cell, but also as a scientific model of differentiation in response to various stimuli.

Chromosome rearrangements are commonly associated with multiple disease states such as cancer. The identification and analysis of these genomic rearrangements have been fundamental for the advancement of research in these diseases. Cell lines are mostly established from such disordered tissues, and in the case of some cultured cells, their genomic constitutions and characteristics continuously alter through passages. Heterogeneity of cells and its derivative cell lines along with different characteristics were also reported in the case of THP-1 [4,6,7]. In the present study, we adopted microarray-based comparative genomic hybridization (aCGH) techniques and attempted to provide a comprehensive and detailed understanding of the genomic aberrations in THP-1 cells.

## Materials and methods

### Genomic DNAs

The THP-1 cell line was subcloned by the limiting dilution technique and 1 clone (#5) was selected for its ability to differentiate relatively homogeneously in response to PMA [8]. THP-1 cells were cultured in RPMI, containing 10% fetal bovine serum (FBS), penicillin/streptomycin, 10 mM HEPES, 1 mM sodium pyruvate and 50 μM 2-mercaptoethanol. Genomic DNA was extracted from 5 × 10^6 ^cells according to the manufacturer's instructions with the illustra GenomicPrep Cells and Tissue DNA Isolation Kit (GE Healthcare UK Ltd., Buckinghamshire, England) and quantified spectrophotometrically. Human Genomic DNA: Female (Promega Corporation, Madison, WI, USA) was purchased as a reference sample.

### Microarray-based CGH Analysis

Oligonucleotide microarray experiment using the Human Genome CGH Microarray Kit 244A (Agilent Technologies, Inc., Santa Clara, CA, USA) was conducted according to manufacturer's protocol (version 5.0). The microarray used for this study was a 1× 244 K slide format printed using Agilent's 60-mer SurePrint technology, and it has 236385 biological features. Its probes span both the coding and noncoding regions for comprehensive genome-wide representation, and the overall median probe spacing is 8.9 kb (7.4 kb in RefSeq genes). THP-1 and human female genomic DNA (1 μg each) were labeled with Cy5 and Cy3, respectively. The hybridized and washed array slide was scanned with an Agilent MicroArray Scanner G2505A (Agilent Technologies, Inc.) and the obtained TIFF image data was processed with Agilent Feature Extraction software (version 9.5.3.1) by the CGH-v4_95_Feb07 protocol (Agilent Technologies, Inc.). Extracted data was analyzed with Agilent DNA Analytics 4.0 software (version 4.0.81) (Agilent Technologies, Inc.) and the Aberration Detection Method 2 (ADM-2) algorithm [9] was used to identify contiguous genomic regions that corresponded to chromosomal aberrations. Following parameters were used in this analysis: Threshold of ADM-2: 6.0; Centralization: ON (Threshold: 6.0, Bin Size: 10); Fuzzy Zero: ON; Aberration Filters: ON (minProbes = 3 AND minAvgAbsLogRatio = 0.5 AND maxAberrations = 10000 AND percentPenetrance = 0); Feature Level Filters: ON (gIsSaturated = true OR rIsSaturated = true OR gIsFeatNonUnifOL = true OR rIsFeatNonUnifOL = true). At a minimum, 3 contiguous suprathreshold probes were required to define a change. To find an obvious homozygous deletion, aberrant regions with a signal log ratio of less than -3.0 were searched. Genomic positions were based on the UCSC March 2006 human reference sequence (hg18) (NCBI build 36.1 reference sequence).

Microarray data generated from this study has been deposited in the Center for Information Biology gene EXpression database (CIBEX) in DNA Data Bank of Japan (DDBJ) (CIBEX accession number: CBX82) [10] and is also available from the Genome Network Platform [11].

## Results and discussion

THP-1 cells were established by Tsuchiya *et al*., and they were reported to have a diploid (46, XY) chromosome number by karyotype analysis [3]. It is important to examine the genomic constitution of these cells in order to interpret the characteristics of THP-1, but there is little information on the whole-genome analysis of THP-1 cells. Odero *et al*. conducted cytogenetic analyses to detect chromosome changes using G-banding, fluorescence *in situ *hybridization (FISH) and spectral karyotyping (SKY) [6]. They reported that these cells have a near-diploid karyotype and contain a number of aberrations. The disadvantage of their techniques, however, was the low-resolution genomic mapping along the chromosome, permitting the identification of only large chromosomal aberrations.

Recently, the microarray-based CGH (aCGH) technique, in particular, the high-density oligonucleotide array-based technique, was established for whole-genome aberration analysis, and this increased the mapping resolution. A high mapping resolution enables precise definition of aberration boundaries and identification of small homozygous deletions, pinpointing the possible locations of tumor-suppressor genes. At present, microarray-based CGH methods focus on detecting copy number changes, but rearrangements, such as balanced translocations or inversions, are not detected by this method.

**Figure 1:**
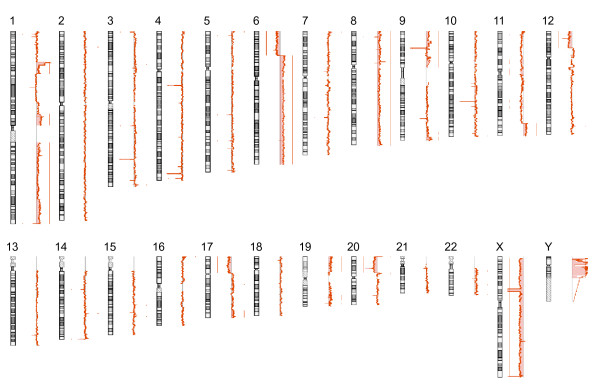
**Chromosomal aberrations detected in the THP-1 cell sample**. Moving averages of signal log ratio for each chromosome (window size = 10 points) are represented as a line plot. Each chromosome is represented on the left. Signal log ratio increases to the right and decreases to the left of centerline. Losses and gains identified by the software are represented by the shaded region and the vertical line.

**Figure 2:**
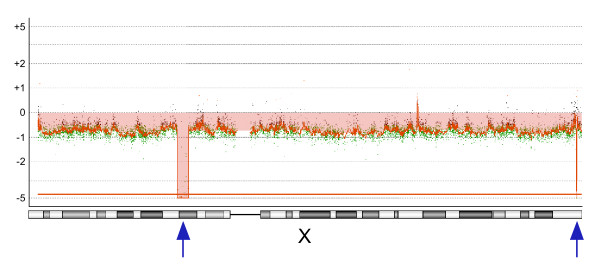
**Regional loss on the chromosome X**. A loss of signals was detected across chromosome X, because the THP-1 cell line derived from a human male patient was compared with the reference human female genome. Two local deletions were also found (arrows). Weighted log ratio data appear as a scatter plot. Green points represent subthreshold values. The identified aberrant region is plotted as a bar graph and shaded in red, and the height of each bar corresponds to the average log ratio for that interval.

Therefore, we adapted an oligonucleotide microarray-based CGH to detect and map regions of DNA gain and loss in case of THP-1 cells. The aCGH experiment revealed 119 aberrant regions in autosomal chromosomes containing nested regions (Figure 1) [see Additional file 1]. Hybridization revealed a gain of chromosome Y and loss of chromosome X in THP-1 cells compared with the reference human female genome; this is because the THP-1 cell line was derived from a human male patient. We did not consider data from the chromosomes X and Y in detail, but we noted the identification of 2 homozygous deletions in chromosome X, namely, chrX: 41631875–44807102 and chrX: 153194224–153211806 (Figure 2). Many genes are located in this region and were probably lost in the THP-1 cell line.

**Figure 3:**
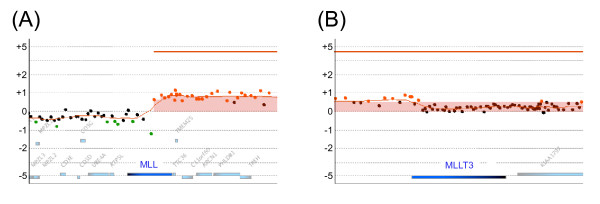
**Breakpoints on the *MLL *and *MLLT3 *genes**. (A) *MLL *gene on chromosome 11. (B) *MLLT3 *gene on chromosome 9. The *MLL *and *MLLT3 *genes had a breakpoint in their gene regions. Weighted log ratio data appear as a scatter plot. Red points represent suprathreshold values. The identified aberrant region is plotted as a bar graph shaded in red, and height of each bar corresponds to the average log ratio for that interval.

Odero *et al*. analyzed THP-1 cells and reported: 49, XY, +der(1)del(1)(p22p36)t(1;12)(p36;q11), del(6)(p21), +del(6)(p21), +8, +der(9)t(9;11)(p22;q23)t(9;11)(q12;q23), -10, der(11)t(9;11)(p22;q23), del(12)(p11), del(17)(p11), der(20)ins(20;1)(p12;p22p36) [13]/48, idem, -del(12)(p11) [7]. We confirmed almost all the reported aberrations of amplifications and deletions, such as trisomy 8 and deletions at 6p, 12p, and 17p, but we didn't find monosomy 10 (Figure 1). Owing to local selective variations, there will probably be differences in the individual cell lines used by different investigators. The differences between individually selected cell lines are presumably responsible for some of the unique results obtained. Translocation t(9;11)(p22;q23) is a recurring chromosomal abnormality in acute myeloid leukemia (AML) cells involving the fusion of 2 genes, *MLL *and *MLLT3*. The genomic *MLL*/*MLLT3 *fusion sequence in THP-1 cells was also reported [6,12]. Odero *et al*. reported duplication of the 3' region of *MLL *and deletion of the *MLLT3 *on the derivative chromosome 9. According to their results, both *MLL *and *MLLT3 *have different copy numbers between the 5' and 3' regions. Although balanced rearrangements are not detected by aCGH methods, we detected amplification in the 3' region of *MLL *and *MLLT3*. We regarded these as breakpoints in the *MLL *and *MLLT3 *genes, because we identified discontinuous copy number changes in *MLL *and *MLLT3 *(Figure 3(A), (B)).

**Figure 4:**
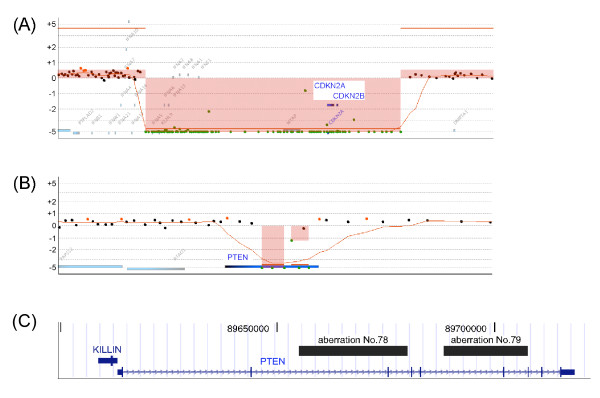
**Regions of homozygous deletion**. (A) Loss of region containing the *CDKN2A *and *CDKN2B *genes. A continuous region containing *CDKN2A *and *CDKN2B *on chromosome 9 was deleted. (B) Partial loss in the *PTEN *gene. *PTEN *lost a part of its gene region. Weighted log ratio data appear as a scatter plot. Green points represent subthreshold values. The identified aberrant regions are plotted as a bar graph shaded in red, and the height of each bar corresponds to the average log ratio for that interval. (C) Deleted regions displayed on the UCSC Genome Browser. Black boxes represent the identified aberration Nos. 78 and 79, and lower row shows schematic structure of the RefSeq genes in this region.

We attempted to find an obvious homozygous deletion, because the loss of tumor-suppressor genes is one reason for immortalization of the cells, and the cells can be effectively characterized by the loss of any other genes. Only 7 regions in all chromosomes were identified, and sum of the extent of these deletions was 4.6 Mb because most of the regions had relatively small deletions (Table 1). Homozygous deletion of the *CDKN2A *and *CDKN2B *genes was previously observed in THP-1 cells similar to that in the case of AML patients [13,14]. Our results confirmed the loss of these genes in the homozygous deletion regions and revealed that the aberration in this region was due to a 965-kb continuous deletion (Figure 4(A)). Localized homozygous deletion in the *PTEN *gene region was also newly identified in THP-1 cells (Figure 4(B), (C)). *PTEN *gene play roles in tumor suppression and maintenance of genomic stability [15]. Somatic mutations in the *PTEN *gene have been identified in a number of cancer cell lines and cancers. The *PTEN *gene has been analyzed in a series of primary acute leukemia and cell lines excluding the THP-1, and it was revealed that a majority of the cell lines carried mutations or hemizygous deletions at this gene locus [16]. Conditional deletion of the *Pten *gene in adult mouse hematopoietic cells led to myeloproliferative disease and leukemia [17]. Analyzing software identified 2 aberrant regions in this gene region, aberration Nos. 78 and 79 [see Additional file 1]. Both deleted regions contained the coding exon of *PTEN *gene (Figure 4(C)). It is presumed that some of the characteristics of THP-1 cells are due to the partial deletion of *PTEN *gene.

**Table 1:** Regions of homozygous deletion in THP-1 cells.

No.	Chr	Cytoband	Position	Size (bp)	Log_2_Ratio	*p*-value	Genes
23	chr3	q26.1	163997028 – 164101976	104949	-3.817441	3.54E-167	
26	chr4	q13.2	69138837 – 69166014	27178	-5.754471	1.76E-28	
28	chr4	q34.3–q35.1	182336611 – 182614113	277503	-6.675501	1.35E-175	
68	chr9	p21.3	21271776 – 22236965	965190	-5.223190	0	IFNA5, KLHL9, IFNA6, IFNA13, IFNA2, IFNA8, IFNA1, IFNE1, MTAP, CDKN2A, CDKN2B, hsa-mir-31
78	chr10	q23.31	89654755 – 89680176	25422	-6.492464	3.11E-67	PTEN
124	chrX	p11.4–p11.3	41631875 – 44807102	3175228	-5.087696	0	CASK, MAOA, MAOB, NDP, EFHC2, FUNDC1, DUSP21, UTX
140	chrX	q28	153194224 – 153211806	17583	-6.345479	5.77E-25	TKTL1

Numbers in the extreme left column correspond with Aberration No. in Additional file 1. Positions were based on the UCSC March 2006 human reference sequence (hg18) (NCBI build 36.1 reference sequence).

**Figure 5:**
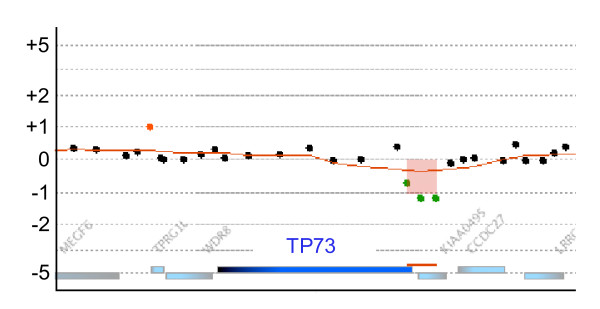
**Deletion in the region containing the *TP73 *gene**. One narrow deletion on chromosome 1 included the *KIAA0495 *and *TP73 *genes. Weighted log ratio data appear as a scatter plot. Green points represent subthreshold values. The identified aberrant region is plotted as a bar graph shaded in red, and the height of each bar corresponds to the average log ratio for that interval.

The high-density oligonucleotide aCGH enabled us to detect more detailed aberrations in comparison with conventional methods. Sixteen aberrations less than 100 kb were found in the identified aberrant regions [see Additional file 1]. For example, we could find only 12-kb of deletion in chr1: 3637672–3650111 containing portions of the *TP73 *and *KIAA0495 *genes (Figure 5). Although there is little information on *KIAA0495*, *TP73 *is known to be a homologue of the *TP53 *gene and it is involved in the regulation of the cell cycle, cell death and development [18]. The number of *TP73 *transcripts was high in AML patient samples as compared with normal cells, and *TP73 *mRNA and protein were strongly expressed in THP-1 cells [19]. Furthermore, AML patients increased expression of the shorter *TP73 *variants, and a particular variant was only expressed in leukemia cell lines [20]. Partial deletion of the *TP73 *locus in THP-1 cells may affect its expression. In a similar fashion, the deletion locus of the *PTEN *gene described above was also small. As 5 of 7 identified homozygous deletions were less than 278 kb and 3 were less than 28 kb (Table 1), we could detect these small deletions using the high-density oligonucleotide aCGH. It is likely that some of these novel narrow aberrations contribute to the characteristics of THP-1 cells.

The FANTOM4/Genome Network Project used THP-1 cells as the model system to understand the transcriptional network underlying growth arrest and differentiation in mammalian cells. A large amount of data, including the genome-wide transcription start site (TSS) and systematic siRNA knockdown of key transcription factors, was generated in this project [8]. We analyzed the identical THP-1 clone used in the FANTOM4/Genome Network Project. The results of this study will provide a useful base for studies in general cell biology, and they would help to precisely understand the data generated by the FANTOM4/Genome Network Project.

## Competing interests

The authors declare that they have no competing interests.

## Authors' contributions

NA analyzed the microarray data and drafted the manuscript. MCH participated in the microarray experiment and a draft for the manuscript. TS prepared experimental samples. HS and TK designed the study and helped to draft the manuscript. All authors read and approved the final manuscript.

## Supplementary Material

Additional file 1 **Aberrant regions in THP-1 cells**. Regions of chromosomal aberration detected by microarray-based CGH analysis. Positions were based on the UCSC March 2006 human reference sequence (hg18) (NCBI build 36.1 reference sequence). Data in the column "Hs_hg18_CNV_20080404" and the column "Hs_hg18_miRNA_20080404" were based on the Database of Genomic Variants (variation.hg18.v4) and the miRBase Sequence Database (Release 10.1), respectively. Click here for file
